# A protecting group-free synthesis of the Colorado potato beetle pheromone

**DOI:** 10.3762/bjoc.9.273

**Published:** 2013-11-06

**Authors:** Zhongtao Wu, Manuel Jäger, Jeffrey Buter, Adriaan J Minnaard

**Affiliations:** 1 Stratingh Institute for Chemistry, University of Groningen, Nijenborgh 7, 9747 AG, Groningen, The Netherlands

**Keywords:** aggregation pheromone, catalysis, Colorado potato beetle, *Leptinotarsa decemlineata*, oxidation, natural product

## Abstract

A novel synthesis of the aggregation pheromone of the Colorado potato beetle, *Leptinotarsa decemlineata,* has been developed based on a Sharpless asymmetric epoxidation in combination with a chemoselective alcohol oxidation using catalytic [(neocuproine)PdOAc]_2_OTf_2_. Employing this approach, the pheromone was synthesized in 3 steps, 80% yield and 86% ee from geraniol.

## Introduction

The Colorado potato beetle *Leptinotarsa decemlineata*, a worldwide pest causing considerable damage in the US annually, has developed resistance to more than 25 insecticides [1–4]. For crop protection, currently several insecticides are used such as the neonicotinoids imidacloprid, thiamethoxam and thiachloprid [1,5], albeit these are expensive and moreover resistance lies in wait [6]. From the standpoint of environmental protection and economics, it is important to reduce the use of insecticides controlling the Colorado potato beetle, and an attractive alternative is to use a pheromone management strategy.

**Figure 1:**
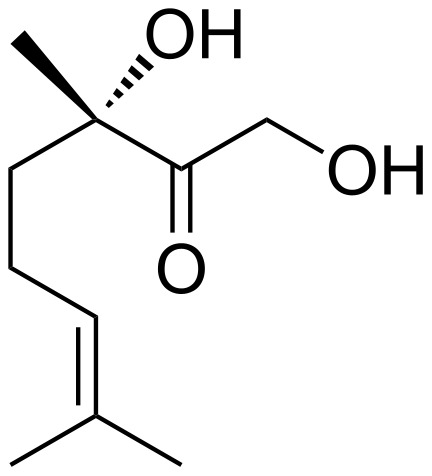
(*S*)-1,3-dihydroxy-3,7-dimethyl-6-octen-2-one (**1**).

An important finding in this connection was the isolation of the male produced aggregation pheromone by Dickens and Oliver et al. in 2002 [7], which was subsequently identified as (*S*)-1,3-dihydroxy-3,7-dimethyl-6-octen-2-one (**1**, Figure 1) [8]. (*S*)-**1** is attractive for both male and female *Leptinotarsa decemlineata* while (*R*)-**1** is inactive or inhibitory, as was demonstrated by the inactivity of the racemate [7]. Since then, (*S*)-**1** has been synthesized by the groups of Oliver [8], Mori [9], Chauhan [10] and very recently by Faraldos [11] respectively, and the first field evaluation of synthetic (*S*)-**1** in a trap crop pest management strategy showed its practical utility [1].

The commercial enantioselective production of chiral pheromones of many pest insects is hampered by prohibitively high costs. The selective introduction of stereocenters and the number of steps are the main reasons. In 2002, Oliver described the first synthesis of both enantiomers of **1** from (*R*)- and (*S*)-linalool, and also the synthesis of its racemate from geraniol, to establish the absolute configuration. Although the approach is elegant, (*S*)-linalool required for natural (*S*)-**1** is not commercially available. In 2005, Mori employing lipase-catalyzed kinetic resolution of (±)-2,3-epoxynerol as the key step, synthesized both (*S*)- and (*R*)-**1** in gram quantities with high ee. In Chauhan’s work, Grignard reaction, oxidation and stereoselective methylation using organometallic reagents are the key steps, affording (*S*)-**1** in high enantiomeric purity and in gram quantities. In all these approaches, however, protection of the primary hydroxy group of the 1,2,3-triol substructure is required for the selective oxidation of the secondary alcohol at C-2. The synthesis by Faraldos runs via epoxidation of fluoronerol, subsequent acetylation of the alcohol and solvolysis.

**Scheme 1:**
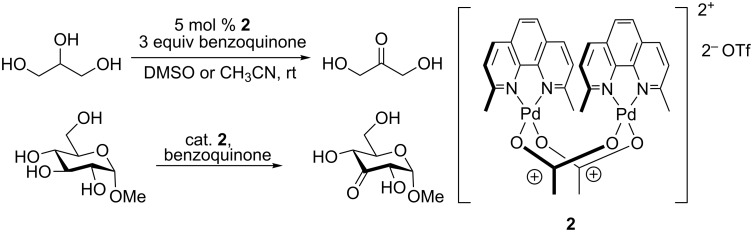
Selective oxidation of glycerol [15] and methyl α-D-glucopyranoside.

**Scheme 2:**
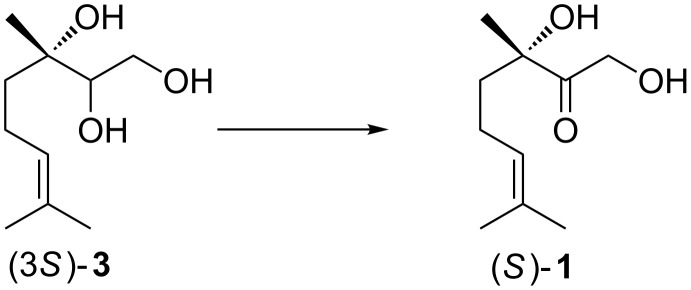
Approach of synthesis of (*S*)-**1**.

In 2010, Waymouth reported the chemoselective, catalytic oxidation of glycerol to dihydroxyacetone (Scheme 1) using catalytic [(neocuproine)PdOAc]_2_OTf_2_ (**2**) in the presence of either benzoquinone or air as the terminal oxidant [12]. More recently, the transformation of unprotected vicinal polyols to α-hydroxy ketones was achieved by regio- and chemoselective oxidation using catalyst **2** [13]. We used this method for the catalytic regioselective oxidation of glycosides (Scheme 1) [14] and expected that the approach might also be applicable in the synthesis of (*S*)-**1**. Triol **3**, with vicinal primary, secondary, and tertiary hydroxy groups should be a suitable substrate for chemoselective oxidation with catalyst **2**, enabling a protecting group-free synthesis of the Colorado potato beetle pheromone (Scheme 2). An additional challenge was the presence of an alkene in the substrate, as the orthogonality of **2**-catalyzed alcohol oxidations with alkenes had not been studied.

In our approach, Sharpless asymmetric epoxidation of readily available geraniol or nerol [16–18] followed by stereoselective ring-opening with water would lead to the desired triol **3**. Subsequently regioselective oxidation of **3** would provide (*S*)-**1** in a concise 3 step route. The resulting synthesis would be interesting also for commercial application, moreover because the oxidation pattern in **1** occurs more widespread in natural products and pharmaceuticals such as in cortisol (hydrocortisone).

## Results and Discussion

**Scheme 3:**
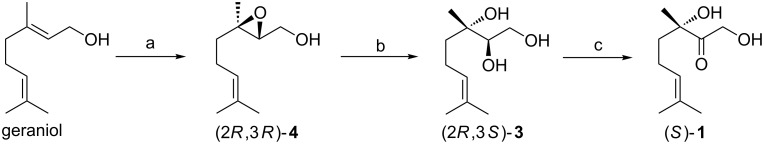
Synthesis of (*S*)-**1** from geraniol. Reagents and conditions: a) D-(−)-diisopropyl tartrate, Ti(OiPr)_4_, *tert*-butyl hydroperoxide, CH_2_Cl_2_, 4 Å MS, –10 to –23 °C, 2 h, 93%, 94:6 er; b) HClO_4_ (70%), THF/water, rt, 30 min, 94%; c) 0.5 mol % **2**, *p*-benzoquinone, CH_3_CN/water, rt, overnight, 91%.

The synthesis of (*S*)-**1** is summarized in Scheme 3. The choice for either geraniol or nerol should have been based on the stereoselectivity of the Sharpless epoxidation. For both reactions, however, varying enantioselectivities had been reported, so both substrates were studied. According to the published procedure, upon treatment of freshly distilled geraniol (Scheme 3) with *tert*-butyl hydroperoxide in the presence of D-(−)-diisopropyl tartrate (DIPT) and Ti(OiPr)_4_ in dry CH_2_Cl_2_ at –10 to –23 °C for 2 h, the desired epoxide (2*R*,3*R*)-**4** was obtained in 93% yield and 88% ee. The ee was determined by HPLC analysis of its corresponding TBDPS ether. This result compares well with the ones reported in the literature: 77–95% yield and 81–95% ee [17–21]. According to Sharpless et al. [18], 5 mol % of Ti(OiPr)_4_ and 7.5 mol % of DIPT were used, so at least 20% excess of tartrate ester in order to obtain the maximum enantiomeric excess. The use of freshly distilled DIPT and Ti(OiPr)_4_ was important to obtain consistently 88% ee. With epoxide (2*R*,3*R*)-**4** at hand, an acid-catalyzed ring-opening reaction was carried out using HClO_4_ in THF/water at room temperature [22]. In the process of ring-opening, close to quantitative inversion of configuration at C-3 takes place [23–24], a result which was confirmed in our research as determination of the ee of both substrate and product shows a slight drop in ee from 88% to 86% (see Supporting Information File 1). Triol (2*R*,3*S*)-**3** was obtained from (2*R*,3*R*)-**4** in high yield by this regio- and stereoselective ring-opening reaction. Subsequently (2*R*,3*S*)-**3** was converted into (*S*)-**1** by treatment with 0.5 mol % of catalyst **2** and benzoquinone in CH_3_CN/water at room temperature. The reaction turned out to be very selective for the secondary alcohol and neither oxidation of the primary alcohol nor of the alkene was observed. (*S*)-**1** was obtained in 91% yield and both ^1^H NMR and ^13^C NMR spectra coincided with those reported in the literature [8–9].

**Scheme 4:**
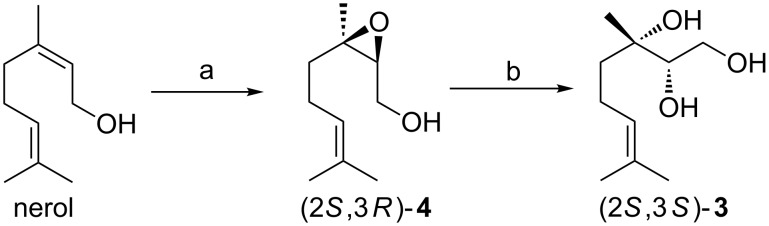
Synthesis starting from nerol. Reagents and conditions: a) L-(+)-diisopropyl tartrate, Ti(OiPr)_4_, *tert*-butyl hydroperoxide, CH_2_Cl_2_, 4 Å MS, −10 to −23 °C, 2 h, 89%, 87:13 er; b) HClO_4_ (70%), THF/H_2_O, rt, 30 min, 92%.

Starting from nerol, Sharpless asymmetric epoxidation afforded the epoxide (2*S*,3*R*)-**4** in a disappointing 74% ee, a result which is nevertheless in accordance with the reported values: 70–94% [25–30] (Scheme 4). Applying the same ring-opening reaction to epoxide (2*S*,3*R*)-**4**, triol (2*S*,3*S*)-**3** was obtained in high yield but a 6% loss in enantiomeric excess was observed (see Supporting Information File 1). Considering these disappointing results using nerol as the starting material, oxidation to (*S*)-**1** was not performed.

## Conclusion

In summary, we have developed an efficient synthesis of the aggregation pheromone of the Colorado potato beetle (*S*)-1,3-dihydroxy-3,7-dimethyl-6-octen-2-one (**1**). Combining Sharpless asymmetric epoxidation, stereoselective epoxide ring-opening and catalytic chemoselective alcohol oxidation with [(neocuproine)PdOAc]_2_OTf_2_ (**2**), (*S*)-**1** was synthesized in 80% overall yield and 86% ee over 3 steps from geraniol. Nerol turned out to be less suitable as starting material as its asymmetric epoxidation provided a lower ee. It has been shown that (*S*)-**1** with an ee of 92% is as active as enantiopure (*S*)-**1** (99% ee), therefore it is probably safe to conclude that the currently obtained 86% ee suffices.

## Supporting Information

File 1 Experimental and spectroscopic details for **1**, **3** and **4**, and determination of the ee of **3** and **4**.

## References

[1] Kuhar T P, Mori K, Dickens J C (2006). Agr Forest Entomol.

[2] Roush R T, Hoy C W, Ferro D N, Tingey W M (1990). J Econ Entomol.

[3] Ioannidis P M, Grafius E, Whalon M E (1991). J Econ Entomol.

[4] Grafius E (1997). J Econ Entomol.

[5] Połeć I (2010). Pestycydy/Pesticides.

[6] Olson E R, Dively G P, Nelson J O (2000). J Econ Entomol.

[7] Dickens J C, Oliver J E, Hollister B, Davis J C, Klun J A (2002). J Exp Biol.

[8] Oliver J E, Dickens J C, Glass T E (2002). Tetrahedron Lett.

[9] Tashiro T, Mori K (2005). Tetrahedron: Asymmetry.

[10] Babu B N, Chauhan K R (2009). Tetrahedron Lett.

[11] Faraldos J A, Coates R M, Giner J-L (2013). J Org Chem.

[12] Painter R M, Pearson D M, Waymouth R M (2010). Angew Chem, Int Ed.

[13] Chung K, Banik S M, De Crisci A G, Pearson D M, Blake T R, Olsson J V, Ingram A J, Zare R N, Waymouth R M (2013). J Am Chem Soc.

[14] Jäger M, Hartmann M, de Vries J G, Minnaard A J (2013). Angew Chem, Int Ed.

[15] Conley N R, Labios L A, Pearson D M, McCrory C C L, Waymouth R M (2007). Organometallics.

[16] Hashimoto M, Harigaya H, Yanagiya M, Shirahama H (1991). J Org Chem.

[17] Katsuki T, Sharpless K B (1980). J Am Chem Soc.

[18] Gao Y, Hanson R M, Klunder J M, Ko S Y, Masamune H, Sharpless K B (1987). J Am Chem Soc.

[19] Nacro K, Baltas M, Escudier J-M, Gorrichon L (1996). Tetrahedron.

[20] Molawi K, Delpont N, Echavarren A M (2010). Angew Chem, Int Ed.

[21] Mohapatra D K, Pramanik C, Chorghade M S, Gurjar M K (2007). Eur J Org Chem.

[22] González I C, Forsyth C J (2000). J Am Chem Soc.

[23] Hanson R M (1984). Tetrahedron Lett.

[24] Corey E J, Ha D-C (1988). Tetrahedron Lett.

[25] Kolb M, Van Hijfte L, Ireland R E (1988). Tetrahedron Lett.

[26] Marshall J A, Trometer J D, Cleary D G (1989). Tetrahedron.

[27] Van Hijfte L, Kolb M (1992). Tetrahedron.

[28] Ray N C, Raveendranath P C, Spencer T A (1992). Tetrahedron.

[29] Mori N, Kuwahara Y, Kurosa K (1996). Bioorg Med Chem.

[30] Underwood B S, Tanuwidjaja J, Ng S-S, Jamison T F (2013). Tetrahedron.

