# Supplementary material for: A protecting group-free synthesis of the Colorado potato beetle pheromone
Source: Beilstein J Org Chem. 2013 Nov 6;9:2374–7. doi: 10.3762/bjoc.9.273 (PMC3869274; doi:10.3762/bjoc.9.273)
Supplement: File 1 — Experimental and spectroscopic details for 1, 3 and 4, and determination of the ee of 3 and 4. [file Beilstein_J_Org_Chem-09-2374-s001.pdf]

Supporting Information

for

A protecting group-free synthesis of the Colorado potato beetle pheromone

Zhongtao Wu, Manuel Jäger, Jeffrey Buter and Adriaan J. Minnaard*

Address: Stratingh Institute for Chemistry, University of Groningen, Nijenborgh 7, 9747 AG, Groningen, The Netherlands.

E-mail: Adriaan J. Minnaard* - a.j.minnaard@rug.nl

* Corresponding author

Experimental and spectroscopic details for 1, 3 and 4, and determination of the ee of 3 and 4.

1. General remarks

^{1}H NMR and ^{13}C NMR spectra were recorded on a Varian AMX400 (400 and 100 MHz, respectively) with $CDCl_3$ as solvent. Chemical shifts were determined relative to the residual solvent peaks ($CHCl_3$, δ = 7.19 ppm for ^{1}H-NMR, δ = 77.0 ppm for ^{13}C NMR). The following abbreviations are used to indicate signal multiplicity: s, singlet; d, doublet; t, triplet; m, multiplet; br, broad. Enantiomeric excesses were determined by chiral HPLC using a Shimadzu LC-20AD HPLC equipped with a Shimadzu SPD-M20A diode array detector and columns (Chiralpak AD-H and OD-H) provided by Daicel corporation, in comparison with the corresponding enantiomers

and racemic mixtures. (2*S*,3*S*)-2,3-Epoxygeraniol was obtained by the same procedure as used for (2*R*,3*R*)-2,3-epoxygeraniol, but using L-(+)-diethyl tartrate. (2*R*,3*S*)-2,3-Epoxynerol was obtained by the same procedure as used for (2*S*,3*R*)-2,3-epoxynerol, but using D-(−)-diisopropyl tartrate (DIPT). Optical rotations were measured on a Schmidt + Haensch polarimeter (Polartronic MH8) with a 10 cm cell (*c* given in g/100 mL) at approx. 20 °C. Thin-layer chromatography (TLC) was performed on Merck TLC Silica gel 60 Kieselguhr F_{254}. Flash chromatography was performed on silica gel Merck Type 9385 230–400 mesh. Geraniol, nerol, D-(−)-diisopropyl tartrate (DIPT), L-(+)-DIPT, titanium tetraisopropoxide and tert-butyl hydroperoxide solution (TBHP) (5~6 M in decane) were purchased from Aldrich. Geraniol, nerol, D-(−)-DIPT, L-(+)-DIPT and titanium tetraisopropoxide were purified by Kugelrohr distillation. The catalyst [(2,9-dimethyl-1,10-phenanthroline)-Pd(μ-OAc)]$_2$(OTf)$_2$ was made according to the literature procedure.[1] 4 Å molecular sieves were dried at 130 °C for 2 d in an oven and heated by a heat gun under vacuum before use.

2. Experimental Section

(2*R*,3*R*)-2,3-Epoxygeraniol (4).[2] To 20 mL of dry CH_2Cl_2 containing 4 Å molecular sieves (2.0 g) were added D-(−)-DIPT (228 mg, 0.97 mmol) and titanium tetraisopropoxide (184 mg, 0.65 mmol) successively at −10 °C under nitrogen. After having added TBHP (3.5 mL, 5~6 M in decane) slowly, the resulted mixture was stirred for an additional 30 min. Then the mixture was cooled to −23 °C by a Cryostat and freshly distilled geraniol (2.0 g, 13.0 mmol) was added over 0.5 h keeping the inner temperature below −20 °C. The mixture was stirred at −23 °C for an additional 2.5 h, and was then quchenched by water (2 mL). The mixture was vigorously stirred for 30 min while allowing to warm to rt. After adding aq NaOH (1.2 mL, 3 M), the mixture was stirred for another 30 min at rt and then filtered over a Büchner funnel under suction. The filtrate was stirred vigorously with 10% aqueous citric acid (6 mL) for 1 h at rt. The organic layer was separated, and the aqueous layer was extracted

with DCM. The combined organic layers were dried over $MgSO_4$, filtered and evaporated in vacuo. The residue was purified by Kugelrohr distillation (120–122 °C, 2 torr) to afford epoxide (2*R*,3*R*)-**4** (2.05 g, 93%) as a colorless oil. $[\alpha]^{20}_{D}$ = +2.3 (*c* 1.32, $CHCl_3$); ^{1}H NMR (400 MHz, $CDCl_3$) δ 5.04-5.00 (m, 1 H), 3.76 (d, *J* = 12.0 Hz, 1 H), 3.61 (dd, *J* = 12.0, 6.4 Hz, 1 H), 2.91 (dd, *J* = 6.8, 4.4 Hz, 1 H), 2.02 (q, *J* = 7.6 Hz, 2 H), 1.84 (br s, 1 H), 1.65-1.54 (m, 1 H), 1.62 (s, 3 H), 1.54 (s, 3 H), 1.44-1.37 (m, 1 H), 1.23 (s, 3 H); ^{13}C NMR (100 MHz, $CDCl_3$) δ 132.1, 123.3, 62.9, 61.4, 61.2, 38.5, 25.6, 23.7, 17.6, 16.7. The spectral data matched with those reported in the literature[2c].

(2*R*,3*R*)-1-tert-Butyldiphenylsilyloxy-2,3-epoxy-3,7-dimethyl-6-octene (5). To a stirred solution of (2*R*,3*R*)-**4** (87 mg, 0.51 mmol) in CH_2Cl_2 (2 mL) were added imidazole (43 mg, 0.63 mmol) and TBDPSCl (0.15 mL, 0.58 mmol) successively at rt. After 5 min, 5 mL of saturated aq NH_4Cl and 5 mL of CH_2Cl_2 were added to the reaction mixture. The organic layer was separated, and the aqueous layer was extracted with CH_2Cl_2. The combined organic layers were dried over $MgSO_4$, filtered and evaporated in vacuo. The residue was purified by column chromatography on silica gel (pentane/Et_2O = 50:1) to afford (2*R*,3*R*)-**5** (188 mg, 90%) as a colorless oil. According to HPLC (Chiral OD-H column, heptane/iPrOH 99.9:0.1, 40 °C, 225 nm) the ee was 88%. Retention time: t_{major} = 23.9 and t_{minor} = 20.4 min. $[\alpha]^{20}_{D}$ = +9.7 (*c* 1.17, $CHCl_3$); ^{1}H NMR (400 MHz, $CDCl_3$) δ 7.62-7.60 (m, 4 H), 7.36-7.29 (m, 6 H), 5.02 (t, *J* = 7.2 Hz, 1 H), 3.73 (dd, *J* = 11.2, 5.2 Hz, 1 H), 3.66 (dd, *J* = 11.2, 5.2 Hz, 1 H), 2.92 (t, *J* = 5.2 Hz, 1 H), 1.99 (q, *J* = 7.6 Hz, 2 H), 1.60-1.32 (m, 2 H), 1.60 (s, 3 H), 1.53 (s, 3 H), 1.06-0.99 (m, 12 H); ^{13}C NMR (100 MHz, $CDCl_3$) δ 135.6, 135.5, 133.5, 133.3, 132.0, 129.7, 127.8, 127.70, 127.69, 123.5, 62.90, 62.86, 60.5, 38.5, 26.8, 25.7, 23.8, 19.2, 17.6, 16.7; HRMS ($C_{26}H_{37}O_2Si$, APCI): calcd. 409.2557, found 409.2556.

(2*R*,3*S*)-3,7-Dimethyl-6-octene-1,2,3-triol (3).[3] To a solution of (2*R*,3*R*)-**4** (200 mg, 1.17 mmol) in THF (5.4 mL) was added dropwise a solution of $HClO_4$ (0.07 mL, 70%) in H_2O (1 mL) at rt. The resulting mixture was stirred for an additional 30 min at rt. Then ethyl acetate (10 mL) and water (3 mL) were added. The organic layer was

separated, and the aqueous layer was extracted with ethyl acetate. The combined organic layers were washed with brine, dried over $MgSO_4$, filtered and evaporated in vacuo. The crude product (2*R*,3*S*)-**3** (206 mg, 94%) was obtained as a colorless oil, sufficiently pure for the next step. $[\alpha]^{20}_D = +5.7$ (*c* 1.02, $CHCl_3$); ^1H NMR (400 MHz, $CDCl_3$) δ 5.04-5.01 (m, 1 H), 3.70-3.61 (m, 5 H), 3.41 (dd, J = 6.4, 3.2 Hz, 1 H), 2.05-1.93 (m, 2 H), 1.61 (s, 3 H), 1.56-1.49 (m, 1 H), 1.54 (s, 3 H), 1.34-1.26 (m, 1 H), 1.14 (s, 3 H); ^{13}C NMR (100 MHz, $CDCl_3$) δ 131.9, 124.1, 76.9, 74.5, 63.1, 37.7, 25.6, 23.2, 22.1, 17.6.

(2*R*,3*S*)-1-tert-Butyldiphenylsilyloxy-3,7-dimethyl-6-octene-2,3-diol (6). To a stirred solution of (2*R*,3*S*)-**3** (50 mg, 0.27 mmol) in CH_2Cl_2 (2 mL) were added imidazole (23 mg, 0.34 mmol) and TBDPSCl (0.08 mL, 0.31 mmol) successively at rt. After 5 min, 5 mL of saturated aq. NH_4Cl and 5 mL of CH_2Cl_2 were added to the reaction mixture. The organic layer was separated, and the aqueous layer was extracted with CH_2Cl_2. The combined organic layers were dried over $MgSO_4$, filtered and evaporated in vacuo. The residue was purified by column chromatography on silica gel (pentane/Et_2O = 3:1) to afford (2*R*,3*S*)-**6** (92 mg, 80%) as a colorless oil. According to HPLC (Chiral AD-H column, heptane/iPrOH 99:1, 40 °C, 230 nm) the ee was 86%. Retention time: t_{major} = 31.5 and t_{minor} = 37.1 min. $[\alpha]^{20}_D = -1.8$ (*c* 0.68, $CHCl_3$); ^1H NMR (400 MHz, $CDCl_3$) δ 7.61-7.58 (m, 4 H), 7.38-7.29 (m, 6 H), 4.97 (t, J = 7.2 Hz, 1 H), 3.77-3.71 (m, 2 H), 3.43-3.40 (m, 1 H), 2.68 (br s, 2 H), 2.03-1.97 (m, 1 H), 1.90-1.83 (m, 1 H), 1.59 (s, 3 H), 1.51-1.45 (m, 1 H), 1.48 (s, 3 H), 1.30-1.24 (m, 1 H), 1.11 (s, 3 H), 0.99 (s, 9 H); ^{13}C NMR (100 MHz, $CDCl_3$) δ 135.55, 135.52, 132.7, 132.6, 131.7, 130.0, 127.873, 127.865, 124.3, 75.8, 73.9, 64.9, 38.1, 26.9, 25.7, 23.2, 22.1, 19.2, 17.6; HRMS ($C_{26}H_{38}O_3SiNa$, APCI): calcd. 449.2482, found 449.2477.

(*S*)-1,3-Dihydroxy-3,7-dimethyl-6-octen-2-one (1). To a suspension of **3** (200 mg, 1.06 mmol) and *p*-benzoquinone (346 mg, 3.20 mmol) in CH_3CN/H_2O (5 mL/0.5 mL) was added [(2,9-dimethyl-1,10-phenanthroline)-Pd(μ-OAc)]$_2$(OTf)$_2$ (5.6 mg, 0.0054 mmol).[4] The resulting mixture was stirred overnight at rt and subsequently filtered over a silica pad. The pad was washed with ethyl acetate and the combined

filtrate was concentrated in vacuo. The residue was purified by column chromatography on silica gel (pentane/EtOAc = 3/1) to afford **1** (180 mg, 91%) as a colorless oil. $[\alpha]^{20}_{D} = +1.6$ (*c* 0.50, $CHCl_3$); ^1H NMR (400 MHz, $CDCl_3$) δ 4.97 (t, J = 7.2 Hz, 1 H), 4.42 (br t, J = 21.6 Hz, 2 H), 2.89 (br s, 2 H), 2.07-1.98 (m, 1 H), 1.88-1.78 (m, 1 H), 1.76-1.63 (m, 2 H), 1.60 (s, 3 H), 1.52 (s, 3 H), 1.30 (s, 3 H); ^{13}C NMR (100 MHz, $CDCl_3$) δ 214.1, 133.3, 122.9, 78.5, 64.6, 39.9, 26.1, 25.6, 22.2, 17.7.

(2*S*,3*R*)-2,3-Epoxynerol (4). (2*S*,3*R*)-**4** was prepared from nerol following a procedure similar to that for geraniol to (2*R*,3*R*)-**4**. $[\alpha]^{20}_{D} = -13.0$ (*c* 1.03, $CHCl_3$); ^1H NMR (400 MHz, $CDCl_3$) δ 5.02 (tt, J = 7.2, 1.2 Hz, 1 H), 3.74 (d, J = 12.0 Hz, 1 H), 3.60-3.55 (m, 1 H), 2.90 (dd, J = 7.2, 4.4 Hz, 1 H), 2.45 (br d, J = 32.0 Hz, 1 H), 2.08-1.99 (m, 2 H), 1.62 (s, 3 H), 1.62-1.57 (m, 1 H), 1.54 (s, 3 H), 1.45-1.37 (m, 1 H), 1.27 (s, 3 H); ^{13}C NMR (100 MHz, $CDCl_3$) δ 132.4, 123.3, 64.4, 61.5, 61.2, 33.1, 25.6, 24.1, 22.1, 17.6. Spectral data matched with those reported in the literature[5].

(2*S*,3*R*)-1-tert-Butyldiphenylsilyloxy-2,3-epoxy-3,7-dimethyl-6-octene (5). (2*S*,3*R*)-**5** was prepared from (2*S*,3*R*)-**4** following a procedure similar to that for (2*R*,3*R*)-**4** to (2*R*,3*R*)-**5**. According to HPLC (Chiral OD-H column, heptane/iPrOH 99.9:0.1, 40 °C, 230 nm) the ee was 74%. Retention time: t_{major} = 31.4 and t_{minor} = 24.7 min. $[\alpha]^{20}_{D} = -7.4$ (*c* 0.76, $CHCl_3$); ^1H NMR (400 MHz, $CDCl_3$) δ 7.61-7.58 (m, 4 H), 7.37-7.32 (m, 6 H), 5.00-4.96 (m, 1 H), 3.81 (dd, J = 8.8, 2.4 Hz, 1 H), 3.74-3.67 (m, 2 H), 2.04 (q, J = 8.4 Hz, 2 H), 1.79-1.65 (m, 2 H), 1.60 (s, 3 H), 1.52 (s, 3 H), 1.40 (s, 3 H), 1.00 (s, 9 H); ^{13}C NMR (100 MHz, $CDCl_3$) δ 135.5, 132.9, 132.8 132.2, 129.92, 129.89, 127.8, 123.3, 77.4, 75.5, 64.3, 40.3, 26.8, 25.64, 25.56, 23.0, 19.2, 17.6. HRMS ($C_{26}H_{36}O_2SiNa$, APCI): calcd. 431.2377, found 431.2372.

(2*S*,3*S*)-3,7-Dimethyl-6-octene-1,2,3-triol (3). (2*S*,3*S*)-**3** was prepared from (2*S*,3*R*)-**4** following a procedure similar to that for (2*R*,3*R*)-**4** to (2*R*,3*S*)-**3**. $[\alpha]^{20}_{D} = -1.1$ (*c* 1.09, $CHCl_3$); ^1H NMR (400 MHz, $CDCl_3$) δ 5.06-5.02 (m, 1 H), 3.68-3.66 (m, 2 H), 3.46-3.44 (m, 1 H), 2.84 (br s, 3 H), 1.99 (apparent q, J = 8.0 Hz, 2 H), 1.61 (s, 3 H), 1.55 (s, 3 H), 1.52-1.50 (m, 1 H), 1.25-1.23 (m, 1 H), 1.10 (s, 3H); ^{13}C NMR (100 MHz, $CDCl_3$) δ 132.0, 124.1, 75.6, 74.5, 63.3, 39.1, 25.7, 22.2, 22.1, 17.6;

HRMS ($C_{10}H_{21}O_3$, APCI): calcd. 189.1485, found 189.1484.

(2*S*,3*S*)-1-tert-Butyldiphenylsilyloxy-3,7-dimethyl-6-octene-2,3-diol (6). (2*S*,3*S*)-**6** was prepared from (2*S*,3*S*)-**3** following a procedure similar to that for (2*R*,3*S*)-**3** to (2*R*,3*S*)-**6**. According to HPLC (Chiral AD-H column, heptane/iPrOH 99:1, 40 °C, 230 nm) the ee was 68%. Retention time: t_{major} = 26.7 and t_{minor} = 20.7 min. $[\alpha]^{20}_{D}$ = −7.2 (*c* 1.09, $CHCl_3$); ^1H NMR (400 MHz, $CDCl_3$) δ 7.61-7.59 (m, 4 H), 7.38-7.31 (m, 6 H), 5.03-5.00 (m 1 H), 3.76-3.68 (m, 2 H), 3.46-3.43 (m, 1 H), 1.94 (dd, *J* = 17.2, 6.8 Hz, 2 H), 1.60 (s, 3 H), 1.52 (s, 3 H), 1.49-1.43 (m, 2 H), 1.02 (s, 3 H), 1.00 (s, 9 H); ^{13}C NMR (100 MHz, $CDCl_3$) δ 135.53, 135.50, 132.7, 132.6, 131.6, 130.0, 127.9, 127.8, 124.3, 75.0, 73.6, 65.0, 39.0, 26.8, 25.7, 22.21, 22.17, 19.2, 17.6; HRMS ($C_{26}H_{38}O_3SiNa$, APCI): calcd. 449.2482, found 449.2478.

^1H NMR data for **(*S*)-1,3-Dihydroxy-3,7-dimethyl-6-octen-2-one (1)**

Assignments	**(*S*)-1**[6]	**(*S*)-1**[7]	**Synthetic (*S*)-1**
	$CDCl_3$<br>500 MHz<br>δ_H (*J*)	$CDCl_3$<br>500 MHz<br>δ_H (*J*)	$CDCl_3$<br>400 MHz<br>δ_H (*J*)
1	4.50 (d, 4.9 Hz)<br>4.48 (d, 4.9 Hz)	4.51 (d, 19.7 Hz)<br>4.47 (d, 19.7 Hz)	4.42 (br t, 21.6 Hz)
2	-	-	-
3	-	-	-
3-Me	1.37 (s)	1.37 (s)	1.30 (s)
4	1.79 (br ddd, 14, 9.8, 6.1 Hz)<br>1.71 (br ddd, 14, 10, 5.8 Hz)	1.79 (br ddd, 14.1, 9.7, 6.0 Hz)<br>1.71 (br ddd, 14.1, 9.7, 6.0 Hz)	1.76-1.63 (m)
5	2.09 (br dt, 14, 6.4 Hz)<br>1.90 (br dt, 14, 6.4 Hz)	2.08 (m)<br>1.89 (m)	2.07-1.98 (m)<br>1.88-1.78 (m)
6	5.04 (br t, 6.4 Hz)	5.04 (tm, 7.2, 1.4 Hz)	4.97 (t, 7.2 Hz)
7-Me	1.58 (s)	1.58 (br s)	1.52 (s)
7-Me	1.67 (s)	1.66 (m)	1.60 (s)
-OH	2.90-2.95 (m)	2.94 (br s)	2.89 (br s)

In our studies, the chemical shift of residual $CHCl_3$ was set to 7.19 ppm whereas in the literature this was 7.26 ppm. Therefore the values of synthetic **1** are consistently 0.07 ppm lower.

^{13}C NMR data for **(*S*)-1,3-Dihydroxy-3,7-dimethyl-6-octen-2-one (1)**

Assignments	**(*S*)-1**[6]	**(*S*)-1**[7]	**Synthetic (*S*)-1**
	$CDCl_3$ 125 MHz δ_H (*J*)	$CDCl_3$ 125 MHz δ_H (*J*)	$CDCl_3$ 100 MHz δ_H (*J*)
1	64.6	64.65	64.6
2	214.2	214.15	214.1
3	78.4	78.47	78.5
3-Me	17.6	17.66	17.7
4	39.9	39.92	39.9
5	22.1	22.16	22.2
6	122.9	122.95	122.9
7	133.2	133.32	133.3
7-Me (*E*)	26.0	26.13	26.1
7-Me (*Z*)	25.6	25.63	25.6

3. References

(1) Conley, N. R.; Labios, L. A.; Pearson, D. M.; McCrory, C. C. L.; Waymouth, R. M. *Organometallics*, **2007**, *26*, 5447–5453.

(2) a) Hashimoto, M.; Harigaya, H.; Yanagiya, M.; Shirahama, H. *J. Org. Chem.* **1991,** *56*, 2299-2311. b) Gao, Y.; Hanson, R. M.; Klunder, J. M.; Ko, S. Y.; Masamune, H.; Sharpless, K. B. *J. Am. Chem. Soc.* **1987,** *109*, 5765-5780. c) Mohapatra, D. K.; Pramanik, C.; Chorghade, M. S.; Gurjar, M. K. *Eur. J. Org. Chem.* **2007,** 5059-5063.

(3) Gonzalez, I. C.; Forsyth, C. J. *J. Am. Chem. Soc.* **2000,** *122*, 9099-9108.

(4) Jäger, M.; Hartmann, M.; de Vries, J. G.; Minnaard, A. J. *Angew. Chem. Int. Ed.* **2013**, *52*, 7809 –7812.

(5) a) Mori, N.; Kuwahara, Y.; Kurosa, K. *Bioorgan. Med. Chem* **1996,** *4*, 289-295. b) Vanhijfte, L.; Kolb, M. *Tetrahedron* **1992,** *48*, 6393-6402.

(6) Tashiro, T.; Mori, K. *Tetrahedron-Asymm.* **2005,** *16*, 1801-1806.

(7) Oliver, J. E.; Dickens, J. C.; Glass, T. E. *Tetrahedron Lett.* **2002,** *43*, 2641-2643.

4. ^{1}H NMR, ^{13}C NMR Spectra and HPLC chromatograms

(2*R*,3*R*)-2,3-Epoxygeraniol (4).

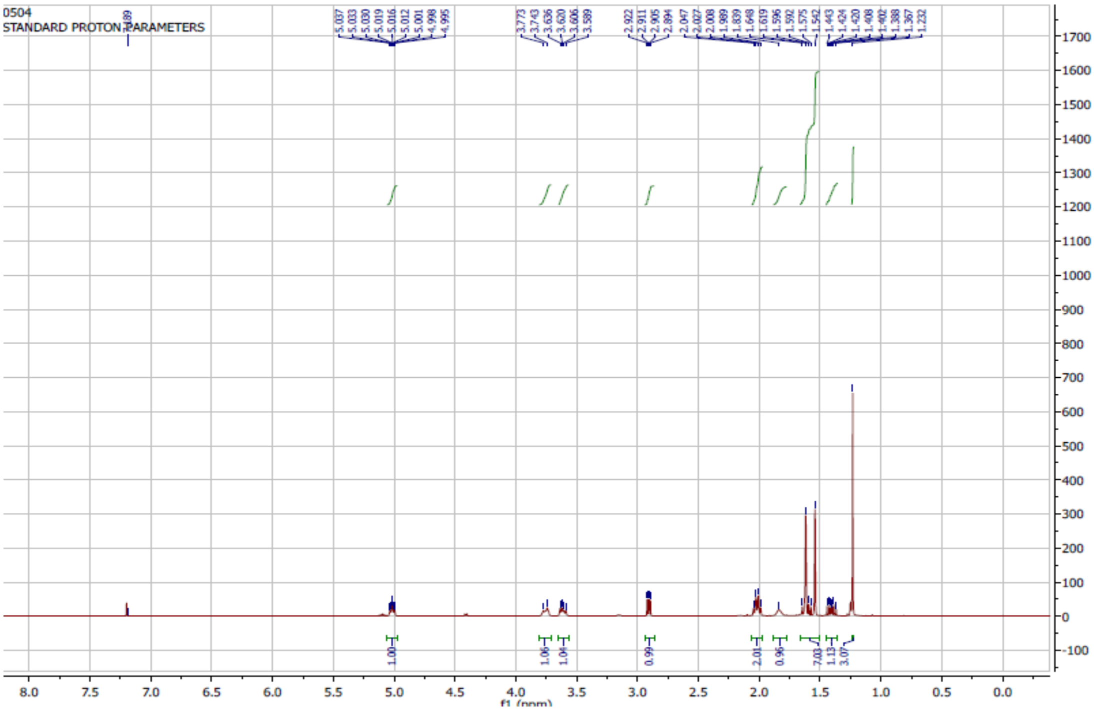

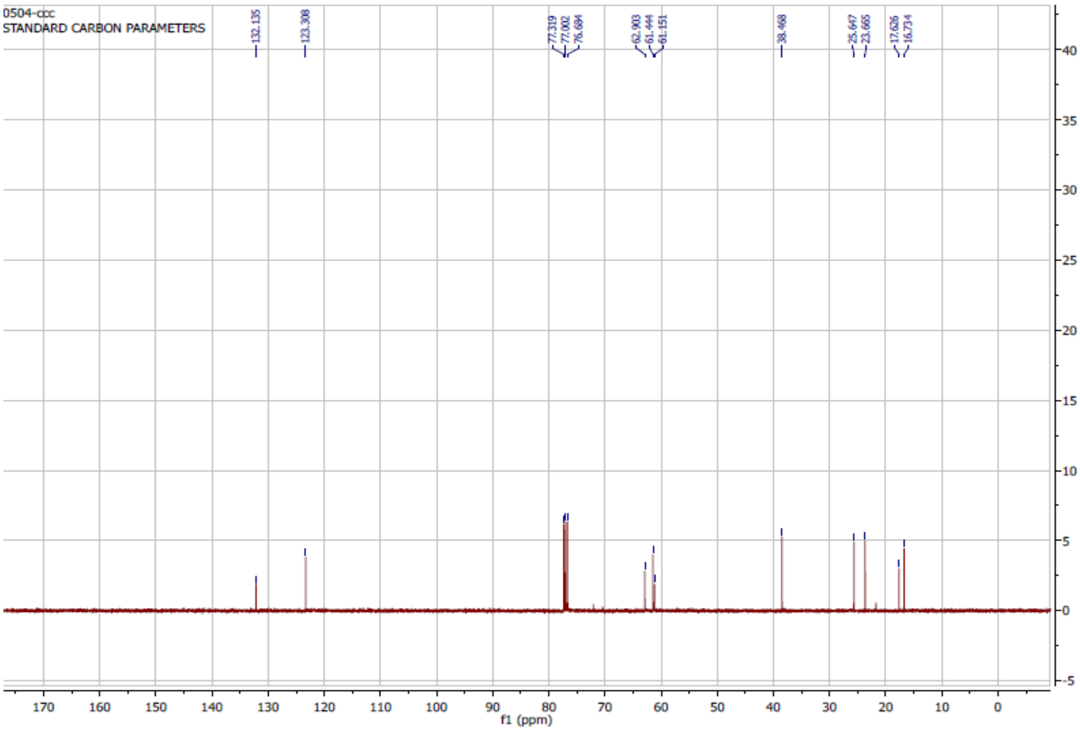

(2*R*,3*R*)-1-tert-Butyldiphenylsilyloxy-2,3-epoxy-3,7-dimethyl-6-octene (5).

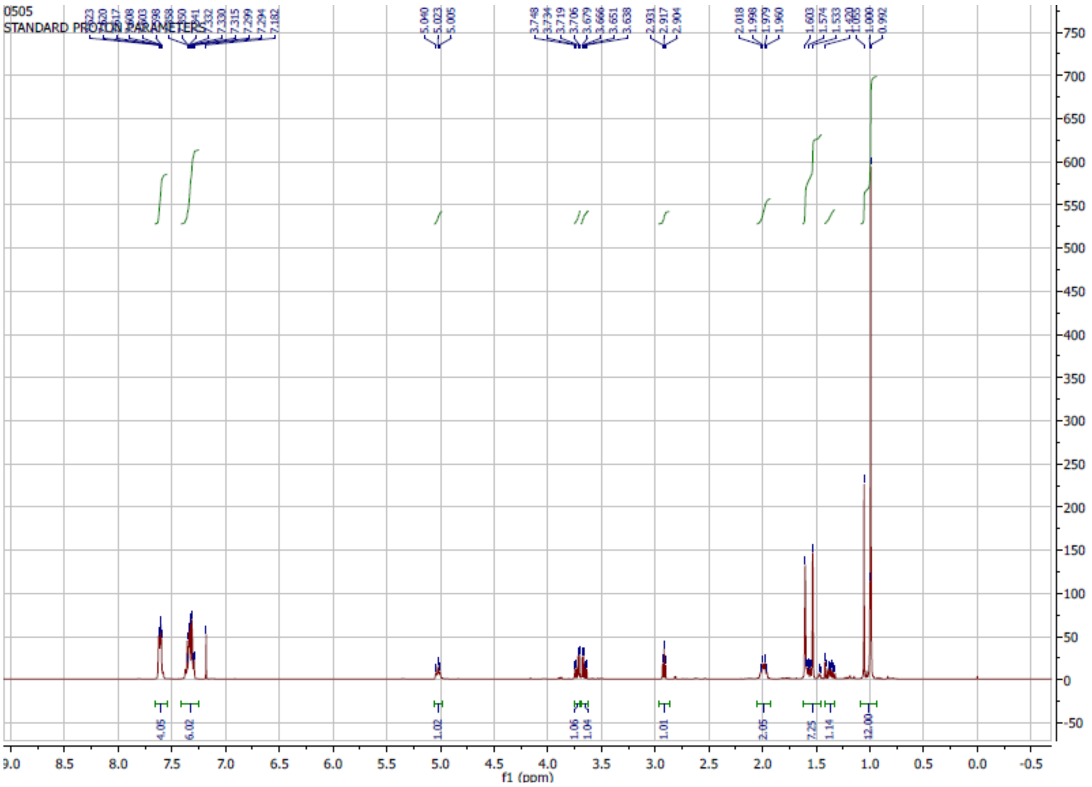

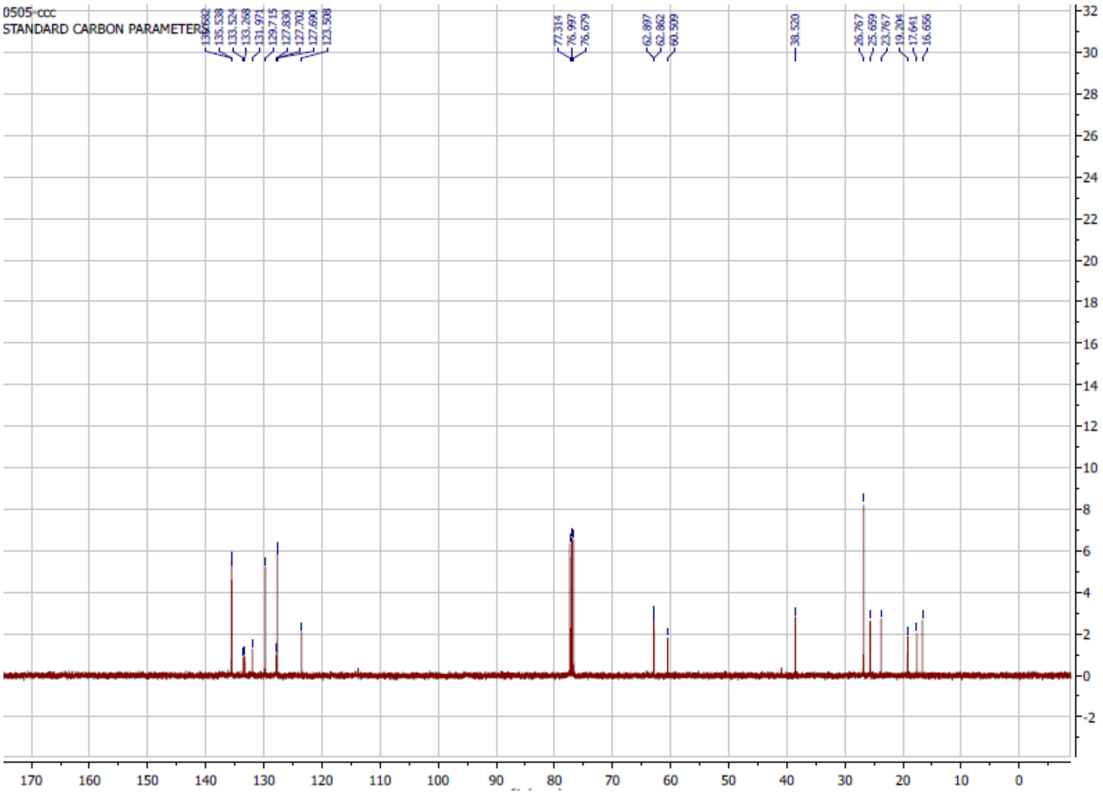

<Chromatogram>

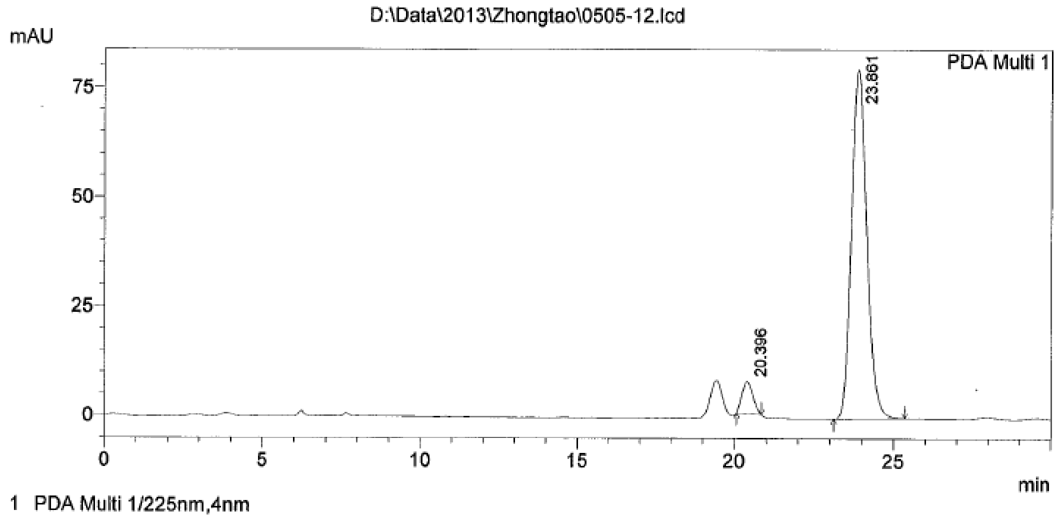

PeakTable

PDA Ch1 225nm

Peak#	Ret. Time	Area	Height	Area %	Height %
1	20.396	177406	7381	6.123	8.465
2	23.861	2720157	79818	93.877	91.535
Total		2897563	87200	100.000	100.000

(2*R*,3*R*)-**5**

<Chromatogram>

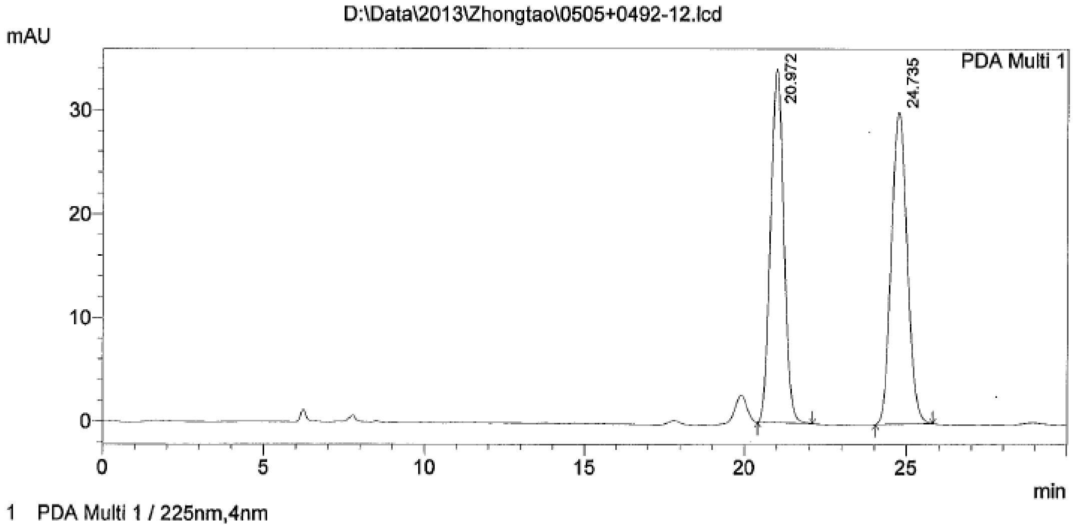

PeakTable

PDA Ch1 225nm

Peak#	Ret. Time	Area	Height	Area %	Height %
1	20.972	962876	34072	48.370	53.131
2	24.735	1027764	30056	51.630	46.869
Total		1990639	64128	100.000	100.000

Mixture of (2*R*,3*R*)-**5** and (2*S*,3*S*)-**5**

(2*R*,3*S*)-3,7-Dimethyl-6-octene-1,2,3-triol (3).

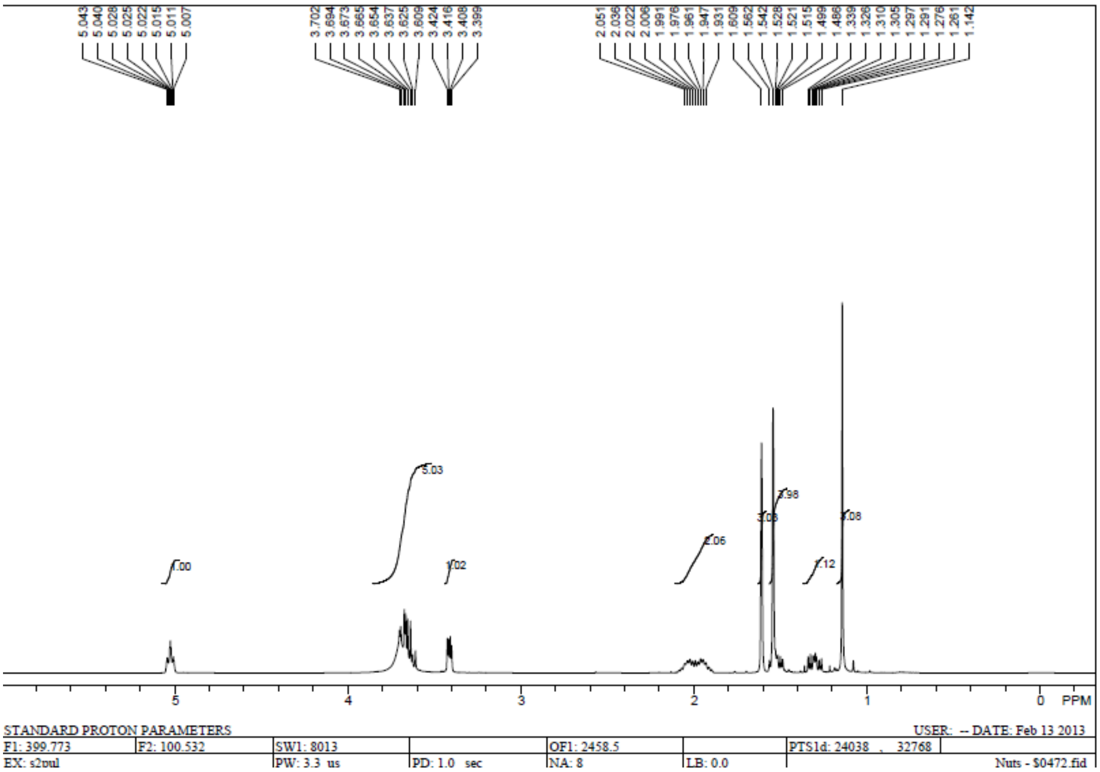

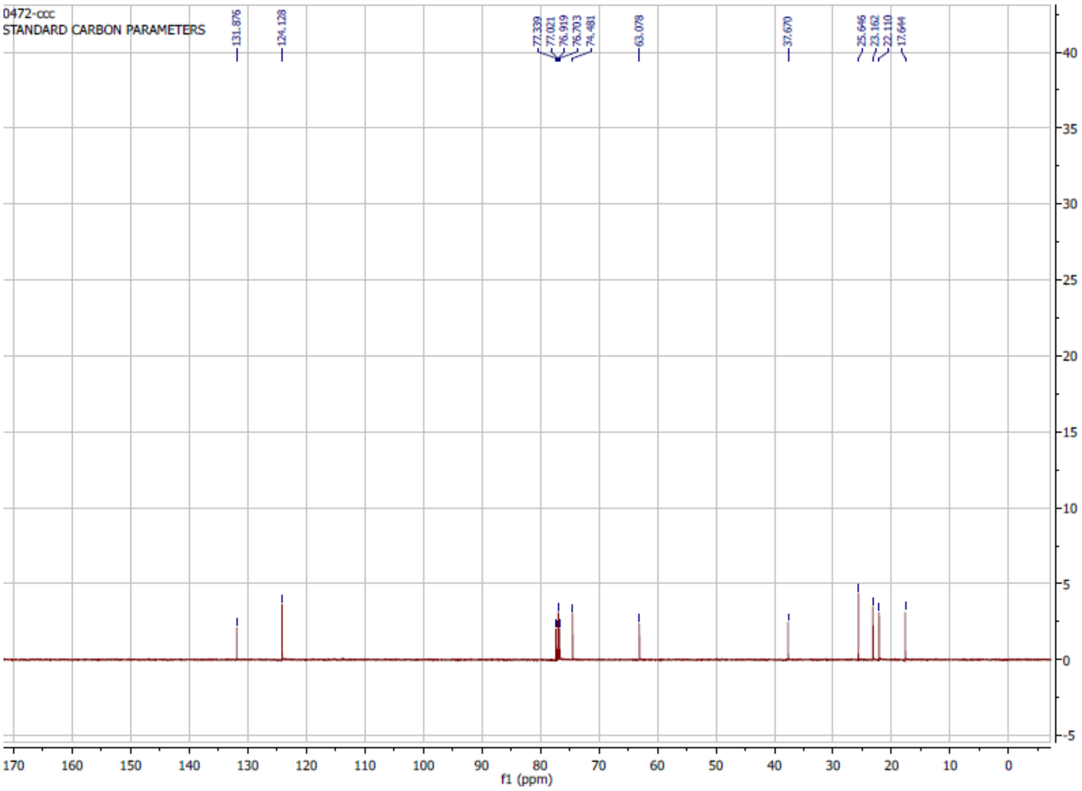

(2*R*,3*S*)-1-*tert*-Butyldiphenylsilyloxy-3,7-dimethyl-6-octene-2,3-diol (6).

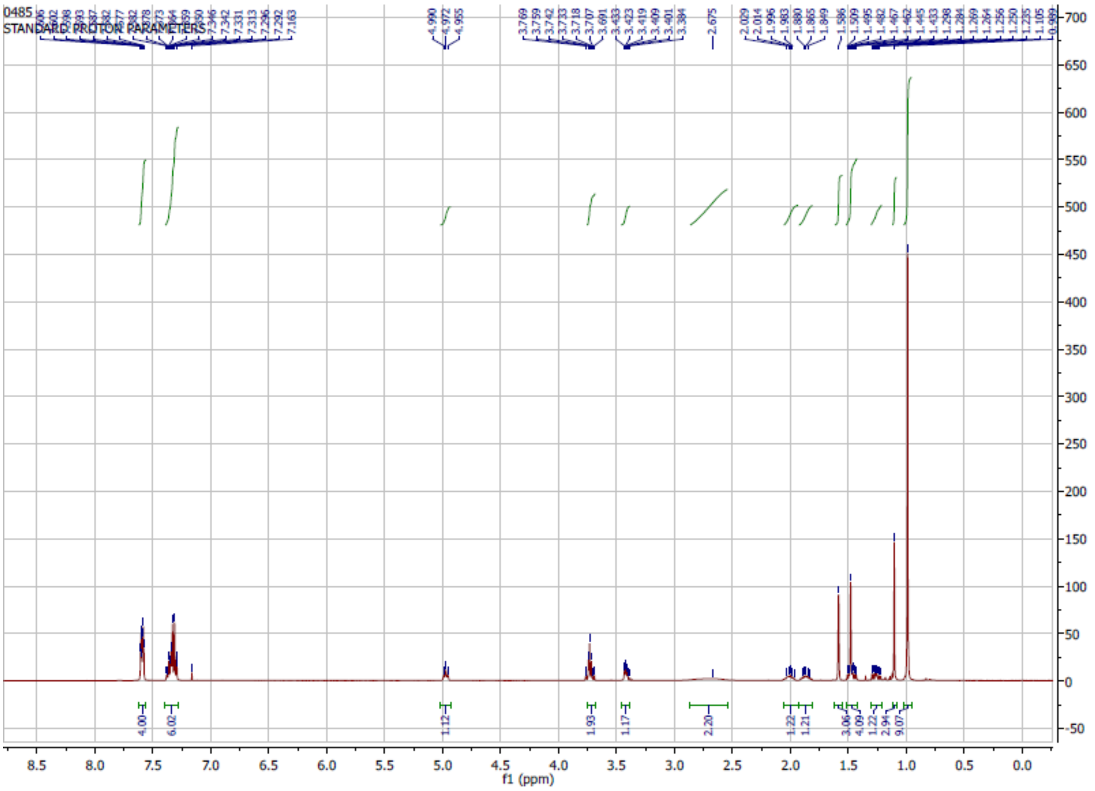

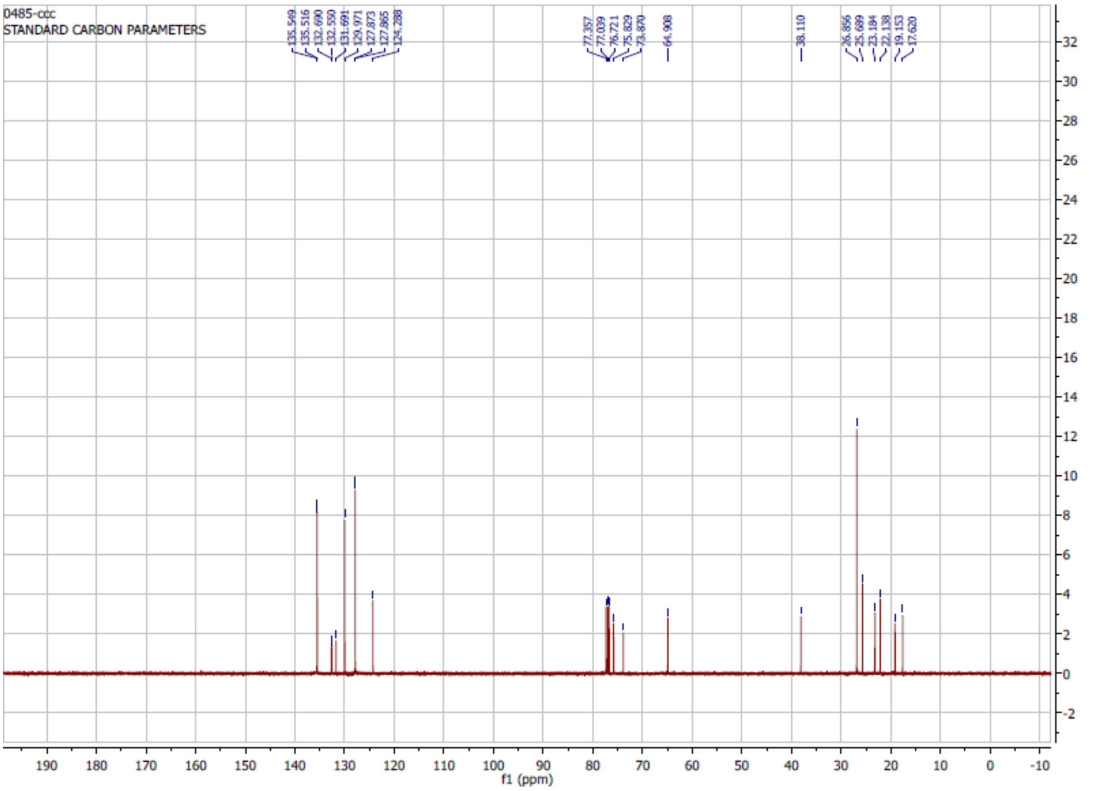

<Chromatogram>

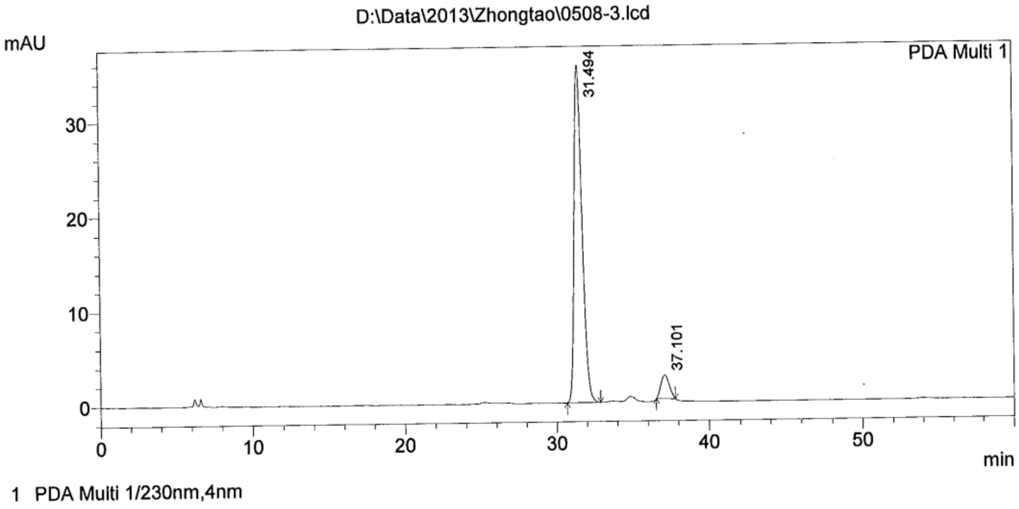

PeakTable

PDA Ch1 230nm

Peak#	Ret. Time	Area	Height	Area %	Height %
1	31.494	1309269	35677	93.235	93.465
2	37.101	94994	2495	6.765	6.535
Total		1404263	38172	100.000	100.000

(2*R*,3*S*)-**6**

<Chromatogram>

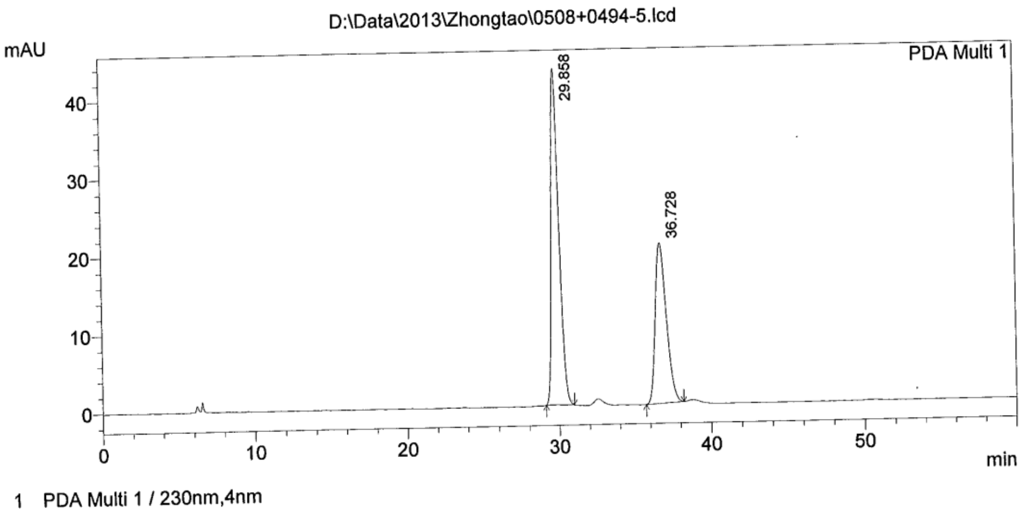

PeakTable

PDA Ch1 230nm

Peak#	Ret. Time	Area	Height	Area %	Height %
1	29.858	1530568	43134	59.645	67.709
2	36.728	1035581	20571	40.355	32.291
Total		2566149	63705	100.000	100.000

Mixture of (2*R*,3*S*)-**6** and (2*S*,3*R*)-**6**

(*S*)-1,3-Dihydroxy-3,7-dimethyl-6-octen-2-one (1).

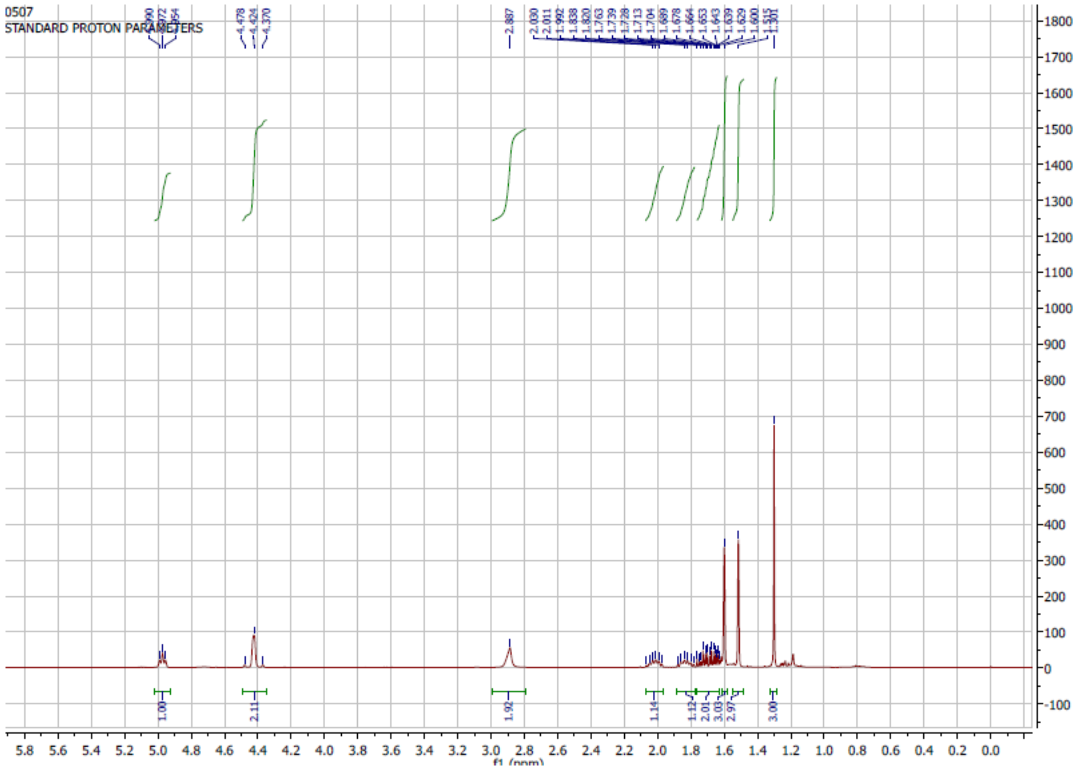

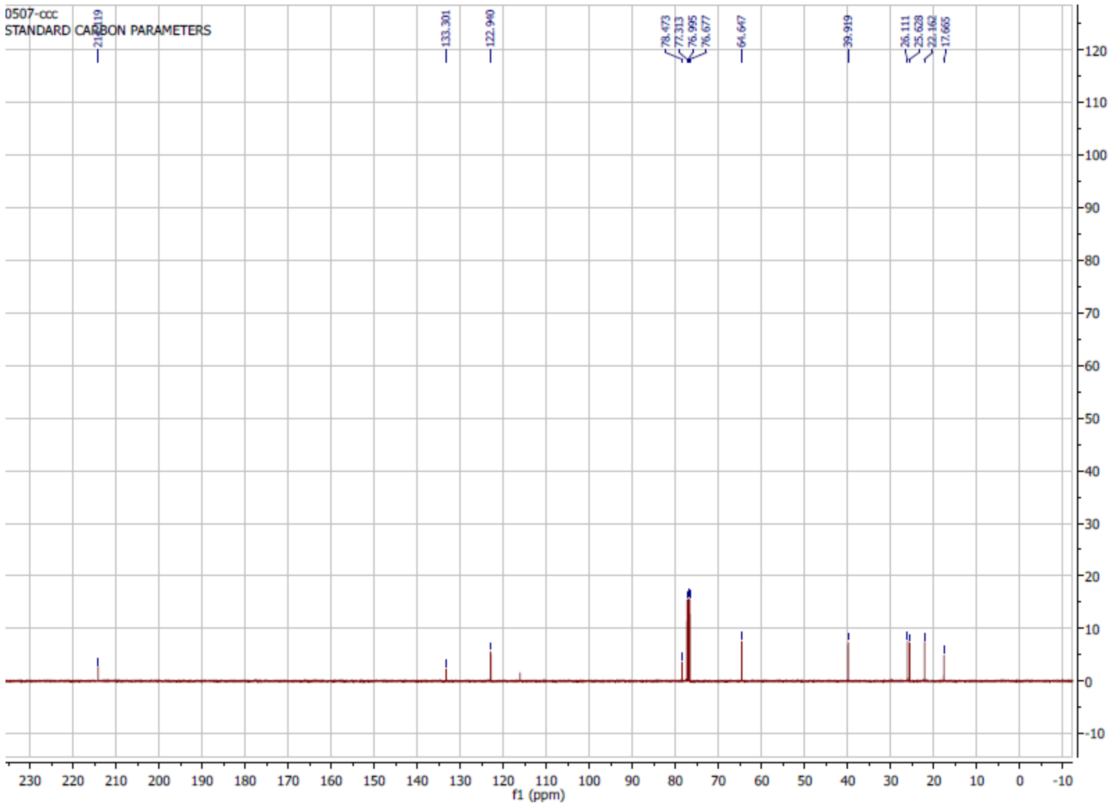

(2*S*,3*R*)-2,3-Epoxynerol (4).

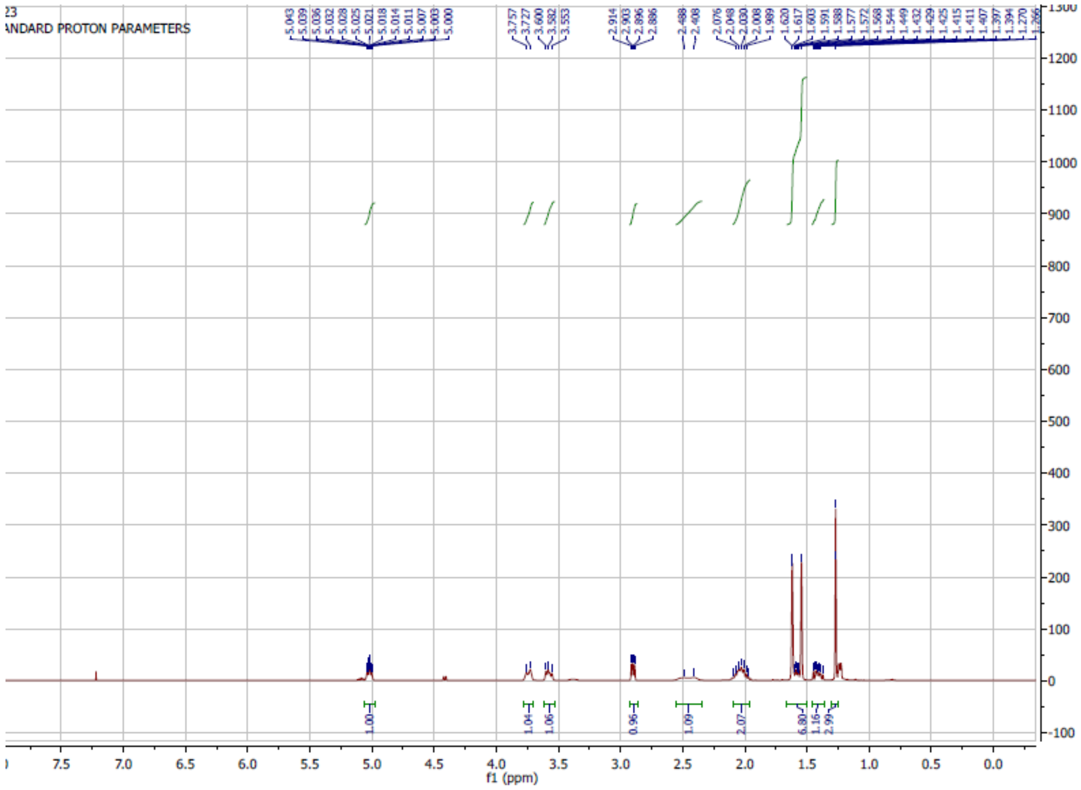

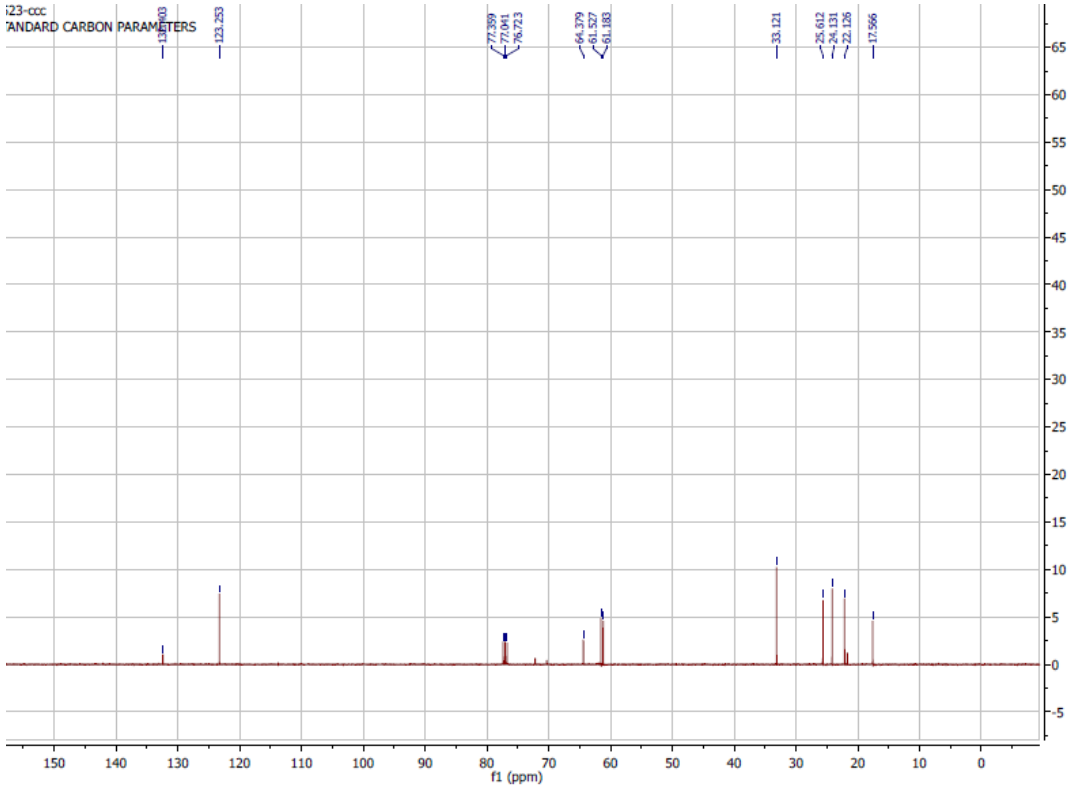

(2*S*,3*R*)-1-tert-Butyldiphenylsilyloxy-2,3-epoxy-3,7-dimethyl-6-octene (5).

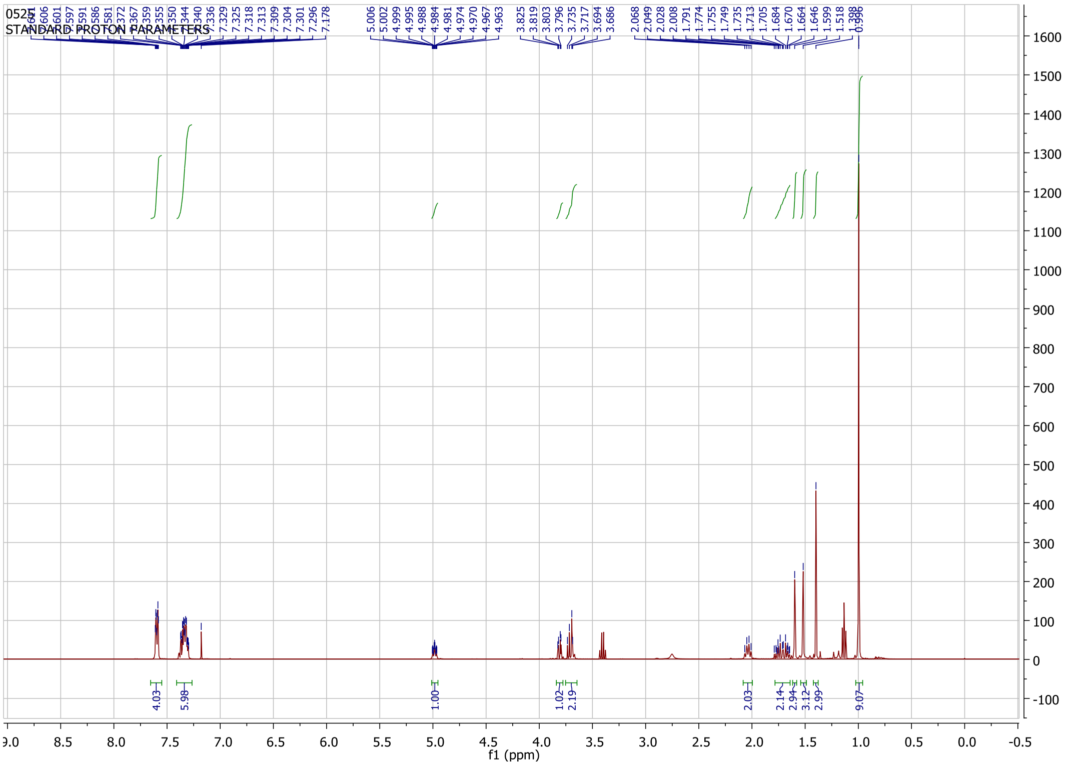

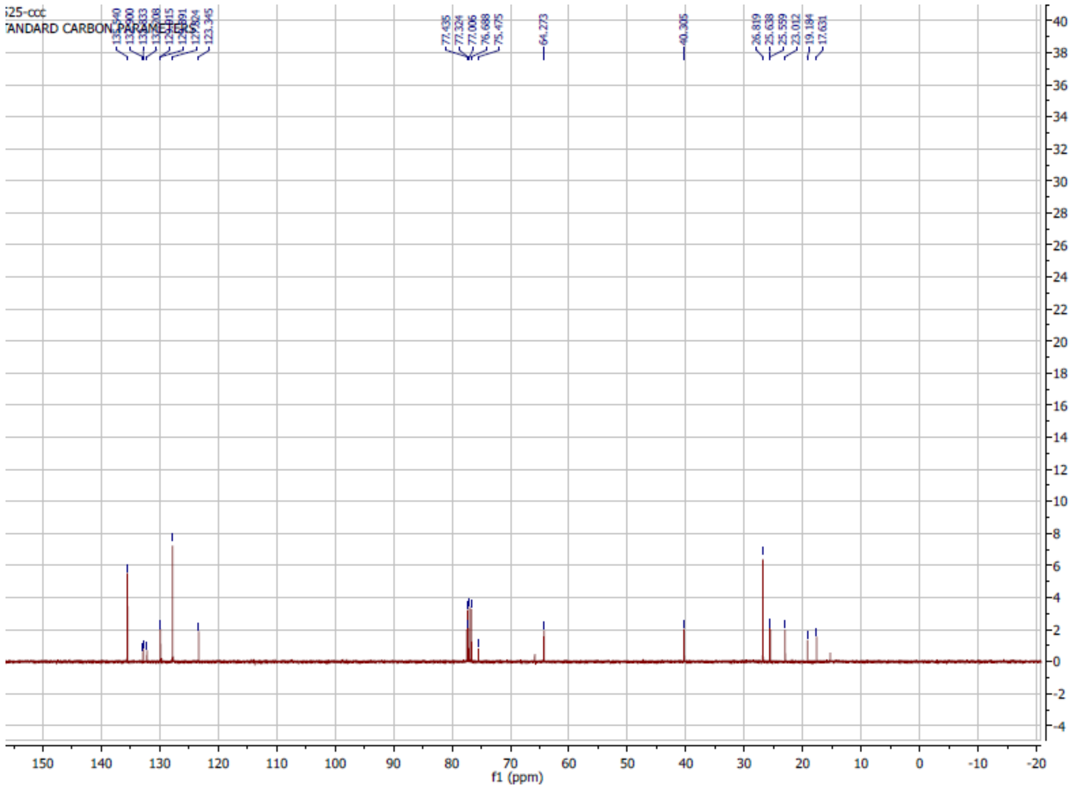

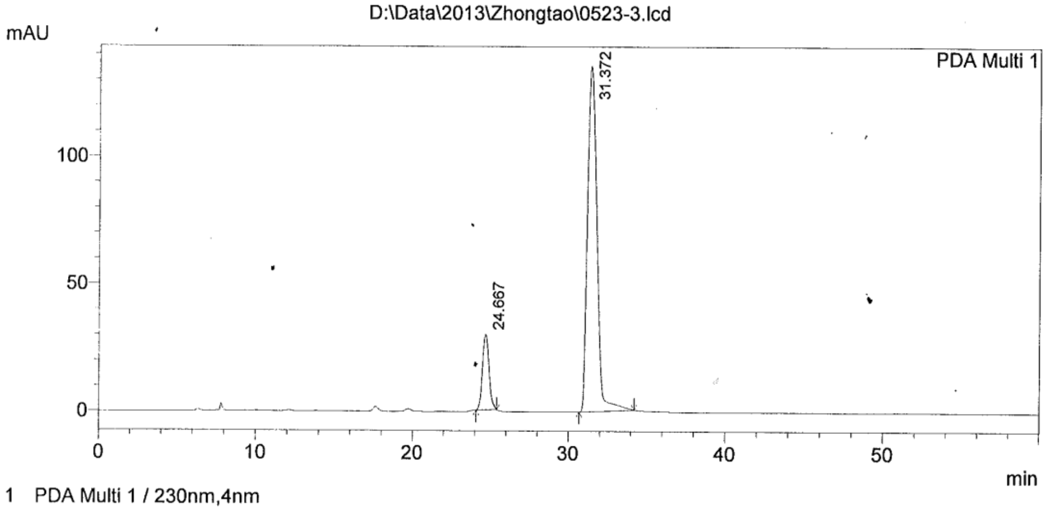

PeakTable

PDA Ch1 230nm

Peak#	Ret. Time	Area	Height	Area %	Height %
1	24.667	868428	29518	13.296	17.904
2	31.372	5662899	135350	86.704	82.096
Total		6531327	164868	100.000	100.000

(2*S*,3*R*)-**5**

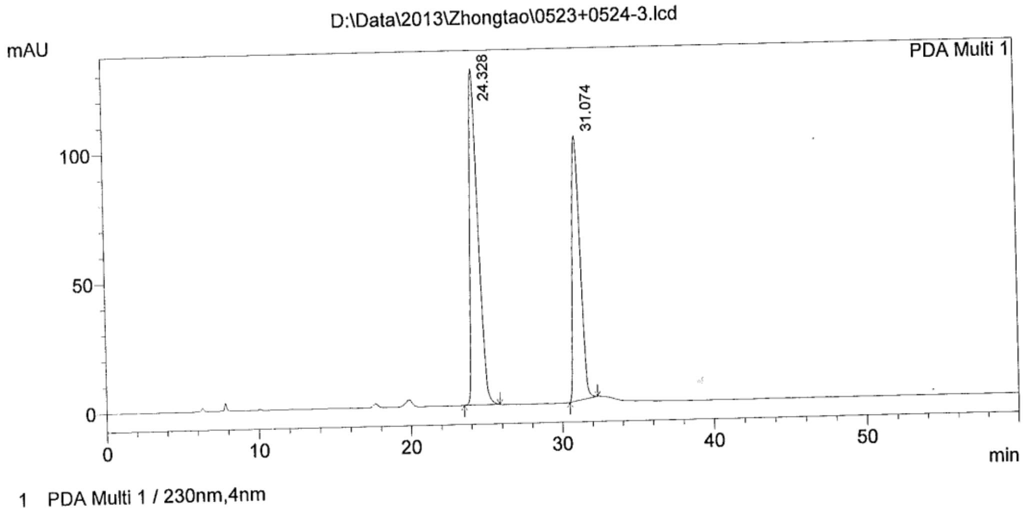

PeakTable

PDA Ch1 230nm

Peak#	Ret. Time	Area	Height	Area %	Height %
1	24.328	4799359	129958	57.312	55.952
2	31.074	3574783	102307	42.688	44.048
Total		8374143	232265	100.000	100.000

Mixture of (2*S*,3*R*)-**5** and (2*R*,3*S*)-**5**

(2*S*,3*S*)-3,7-Dimethyl-6-octene-1,2,3-triol (3).

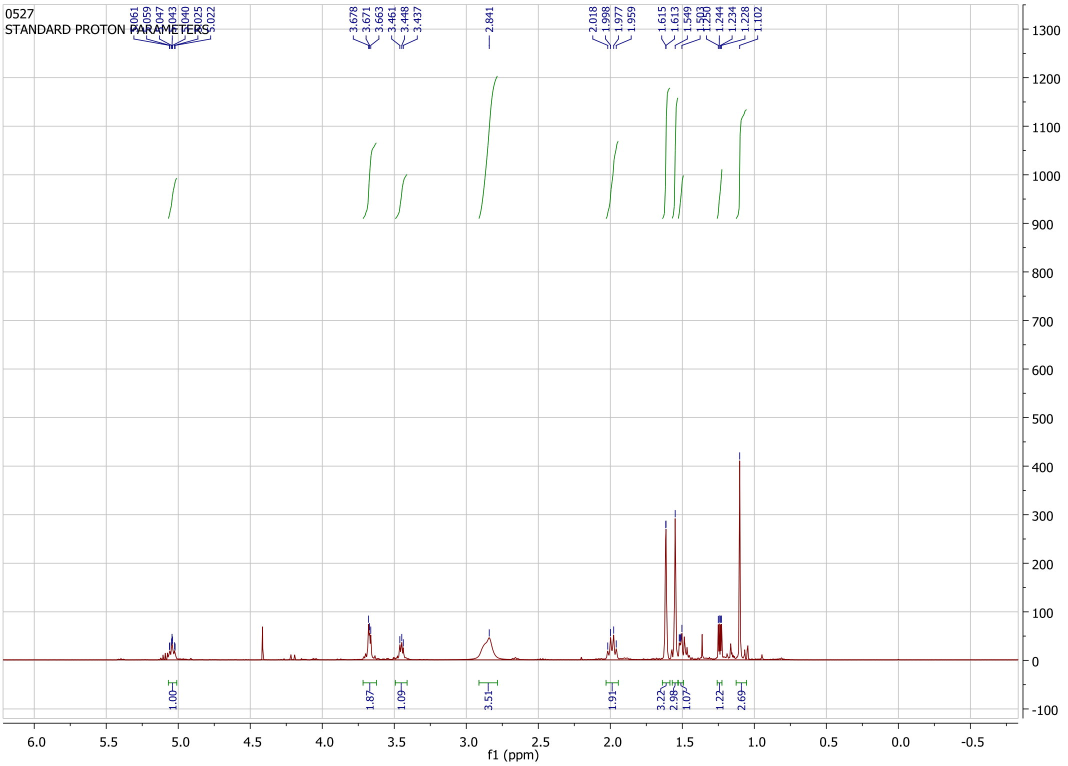

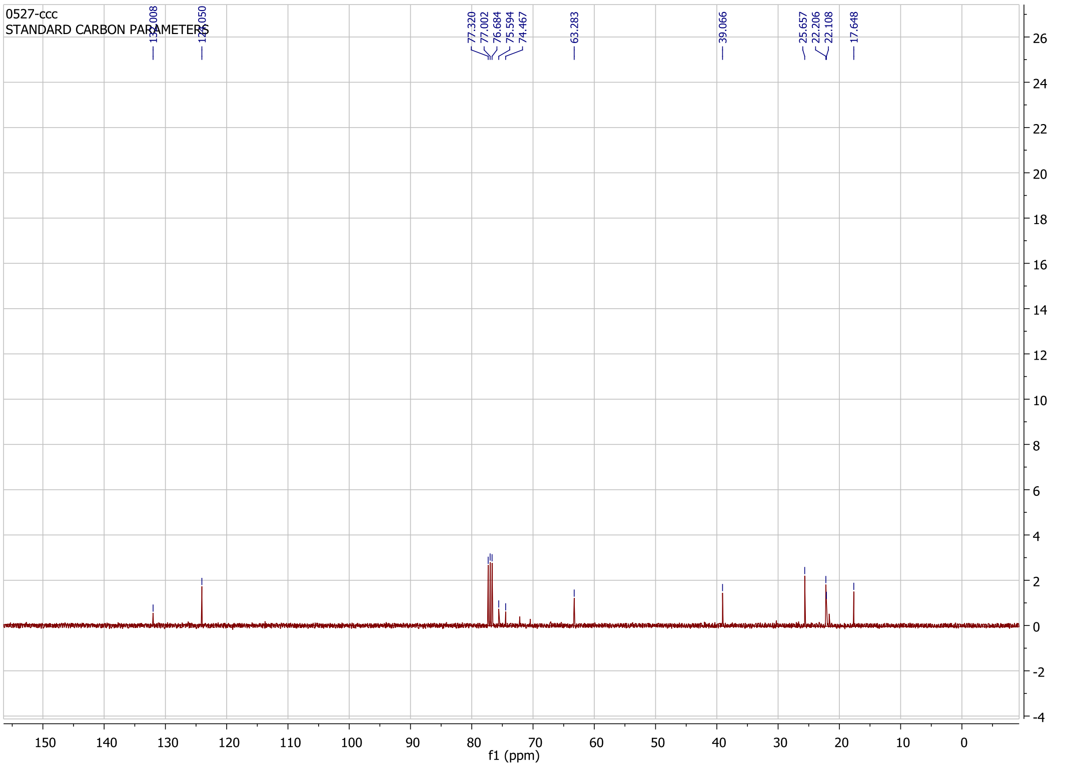

(2*S*,3*S*)-1-tert-Butyldiphenylsilyloxy-3,7-dimethyl-6-octene-2,3-diol (6).

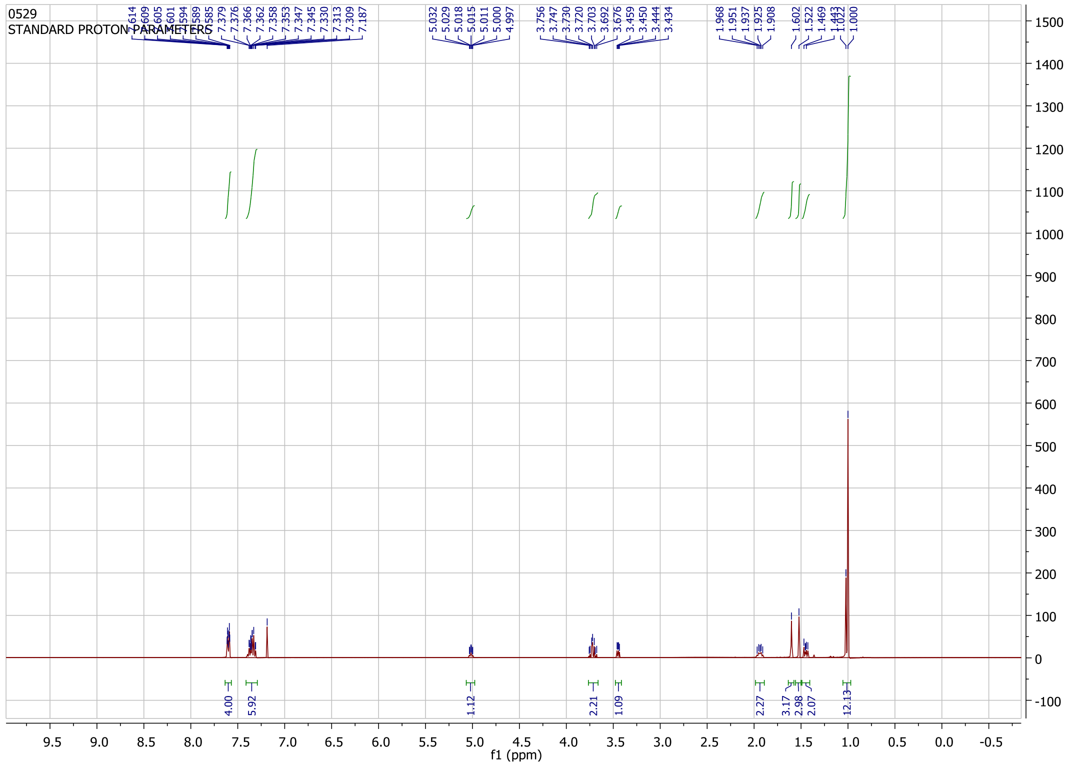

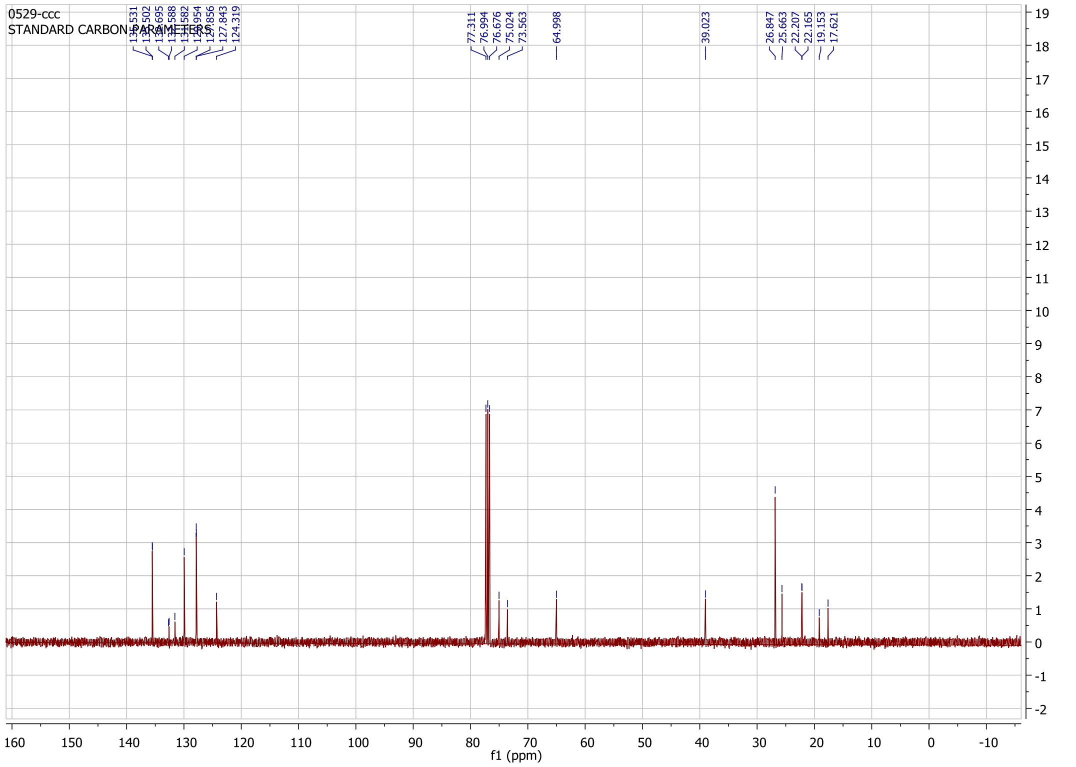

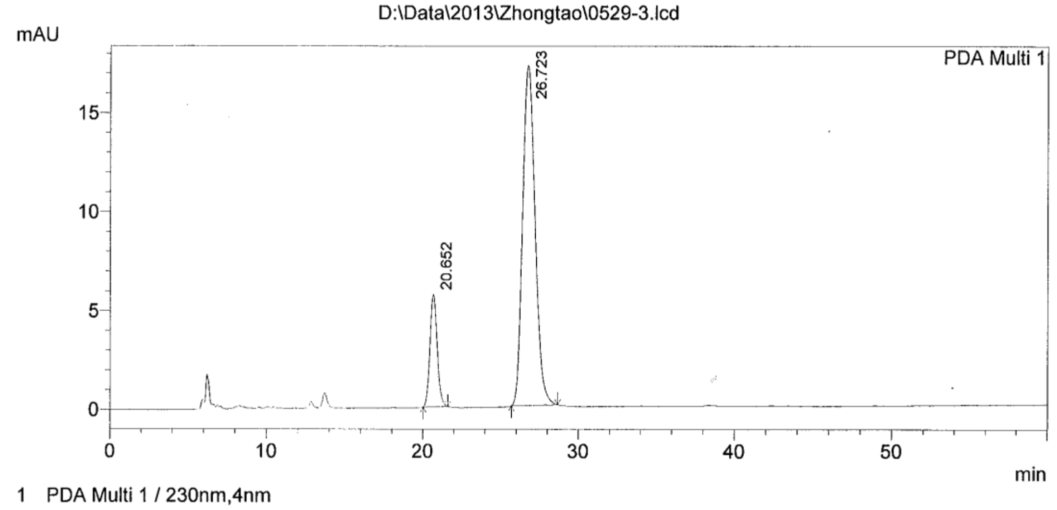

PeakTable

PDA Ch1 230nm

Peak#	Ret. Time	Area	Height	Area %	Height %
1	20.652	181841	5662	15.916	24.796
2	26.723	960680	17173	84.084	75.204
Total		1142522	22835	100.000	100.000

(2*S*,3*S*)-**6**

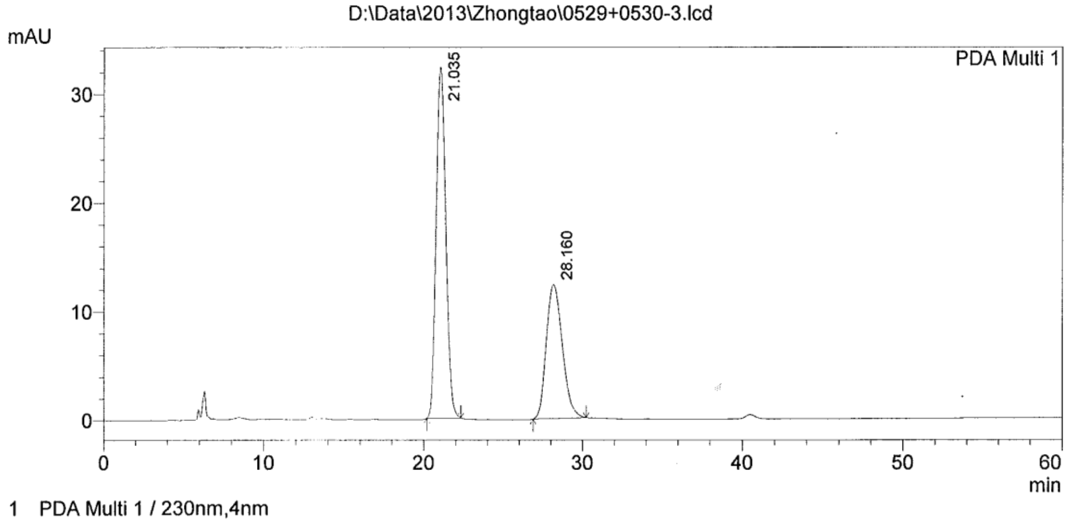

PeakTable

PDA Ch1 230nm

Peak#	Ret. Time	Area	Height	Area %	Height %
1	21.035	1364417	32302	61.067	72.391
2	28.160	869882	12319	38.933	27.609
Total		2234299	44621	100.000	100.000

Mixture of (2*S*,3*S*)-**6** and (2*R*,3*R*)-**6**
